# Mapping global changes in nuclear cytosine base modifications in the early mouse embryo

**DOI:** 10.1530/REP-15-0207

**Published:** 2016-02

**Authors:** Y Li, Michelle K Y Seah, C O'Neill

**Affiliations:** Centre for Developmental and Regenerative Medicine, Kolling Institute for Medical Research, Sydney Medical School, University of Sydney, Sydney, New South Wales, 2065, Australia

## Abstract

Reprogramming epigenetic modifications to cytosine is required for normal embryo development. We used improved immunolocalization techniques to simultaneously map global changes in the levels of 5′-methylcytosine (5meC) and 5′-hydroxymethylcytosine (5hmC) in each cell of the embryo from fertilization through the first rounds of cellular differentiation. The male and female pronuclei of the zygote showed similar staining levels, and these remained elevated over the next three cell cycles. The inner cells of the morula showed a progressive reduction in global levels of both 5meC and 5hmC and further losses occurred in the pluripotent inner cell mass (ICM) of the blastocyst. This was accompanied by undetectable levels of DNA methyltransferase of each class in the nuclei of the ICM, while DNA methyltransferase 3B was elevated in the hypermethylated nuclei of the trophectoderm (TE). Segregation of the ICM into hypoblast and epiblast was accompanied by increased levels in the hypoblast compared with the epiblast. Blastocyst outgrowth *in vitro* is a model for implantation and showed that a demethylated state persisted in the epiblast while the hypoblast had higher levels of both 5meC and 5hmC staining. The high levels of 5meC and 5hmC evident in the TE persisted in trophoblast and trophoblast giant cells after attachment of the blastocyst to the substratum *in vitro*. This study shows that global cytosine hypomethylation and hypohydroxymethylation accompanied the formation of the pluripotent ICM and this persisted into the epiblast after blastocyst outgrowth, and each differentiated lineage formed in the early embryo showed higher global levels of 5meC and 5hmC.

## Introduction

It is considered that epigenetic information is encoded within the structure and conformation of chromatin. This provides for lineage specific, mitotically heritable patterns of gene expression that define the ontological fate and function of a cell. One of the most extensively investigated epigenetic signatures is the 5′-methylation of cytosine bases (5meC) within cytosine–guanine dinucleotides (CpG) in the genome (Klose & Bird 2006, Ooi *et al*. 2009). 5meC can be further modified by the actions of the methylcytosine dioxygenase (TET) class of enzymes to progressively form 5′-hydroxymethylcytosine (5hmC), 5′-formylcytosine, and 5′-carboxylcytosine. Each of these TET-mediated modifications may provide its own epigenetic information to the cell, with 5hmC being the most prevalent and has received the most experimental attention (Ito *et al*. 2010, Kubiura *et al*. 2012).

Reprogramming the patterns of 5meC and 5hmC modification of the genome is widely considered to be an important part of early embryonic development. A range of stresses to the early embryo can perturb the normal patterns of epigenetic reprogramming (Mahsoudi *et al*. 2007, Morgan *et al*. 2008, Watkins *et al*. 2008) causing an increased propensity for post-natal homeostatic instability.

Base-level analysis has shown that the paternally- and maternally-derived genomes in the zygote have similar global levels of cytosine methylation, with a small reduction over subsequent cell cycles, while the cells of the inner cell mass (ICM) show a major loss of methylation in most classes of genetic elements, with the exception of numerous CpG islands and 5′-UTRs (Smith *et al*. 2014, Wang *et al*. 2014). To date, there has been relatively limited analysis of the trophectoderm (TE) of the blastocyst. After implantation (E6.5–E7.5) the embryo as a whole becomes hypermethylated compared to the pre-implantation ICM (Wang *et al*. 2014); but by this stage the embryo is composed of several cell types so whole embryo analysis does not reveal whether this hypermethylation affects each of the emerging cell lineages. The small size, increasing structural complexity, and the relatively low sensitivity of basal-level analytical tools has not allowed detailed base-level analysis of each of these cell lineages as they emerge.

Immunolocalization is an important tool in defining changes in methylation in the embryo, having the advantage of being the only method capable of assessing global changes in the levels of the various forms of cytosine modification within the same cell. This provides a particular advantage in tracing changes in methylation within the embryo as it gains structural complexity with development. However, DNA is a highly complex and dynamic structure, and this complexity provides many opportunities for the masking of antigens from antibody detection. Previous studies have pointed to a round of global active demethylation of the paternally-derived genome to levels far below that of the maternal DNA in the zygote (Mayer *et al*. 2000, Santos *et al*. 2002) and high levels of 5meC immunostaining in the ICM (Santos *et al*. 2002). However, these findings are not consistent with recent base-level analyses, which have shown modest loss of methylation from the male DNA, bringing it to a level similar to that of maternally-derived DNA, and low global levels of methylation in the ICM compared to earlier stages (Smith *et al*. 2014, Wang *et al*. 2014). Methods for improved solvent exposure and staining of the 5meC and 5hmC epitopes under equilibrium conditions were recently described (Li & O'Neill 2012, 2013). These revealed higher and more stable levels of methylation than expected in the early embryo and showed that earlier reports of pervasive global active demethylation were accounted for by progressive masking of the 5meC antigen during zygotic maturation. The faithful exposure of the antigen showed similar levels of methylation in both pronuclei in the zygote and also showed that this methylation persisted over the following cell cycles.

The aim of the present study was to use this new immunolocalization method that reliably retrieves epigenetic epitopes in order to extend the mapping of the global patterns of expression of these two most prominent epigenetic modifications of cytosine in each cell of the early embryo and in each of the first lineages formed during development. The results show that the pluripotent ICM and epiblast are significantly hypomethylated and hypohydroxymethylated and that each of the differentiated lineages formed during early development are characterized by marked changes in the relative levels of each of these modifications.

## Materials and methods

### Animals

The use of animals was in accordance with the Australian Code of Practice for the Care and Use of Animals for Scientific Purposes and was approved by the Royal North Shore Hospital Animal Care and Ethics Committee. Hybrid (C57BL/6j X CBA/He) mice were used in all experiments. Animals were housed and bred in the Kearns Facility, Kolling Institute, St Leonards, NSW, Australia. All animals were under 12 h light: 12 h darkness cycle and had access to food and water *ad libitum*. Six-week-old female mice were superovulated by intraperitoneal injection of 5 IU equine chorionic gonadotrophin (Folligon, Intervet International, Boxmeer, The Netherlands) followed 48 h later by 5 IU hCG (Chorulon, Intervet International) and then were paired with male mice of proven fertility. Pregnancy was confirmed by the presence of a copulation plug the following morning (day 1).

### Mouse embryo collection and culture

Different stage mouse embryos were collected from the reproductive tract (Fresh) in Hepes buffered human tubal fluid medium (Hepes-HTF) (O'Neill 1997) at times after hCG (hour post-hCG) and were immediately fixed for analysis, unless otherwise indicated in specific experiments. In one experiment embryos were produced by IVF as previously described (O'Neill 1997). IVF embryos were cultured in KSOM media supplemented with amino acids (Ho *et al*. 1995). All components of the media were tissue culture grade (Sigma–Aldrich Co.) and contained 3 mg/ml BSA (Sigma) (KSOM+AA). For those collected directly from the reproductive tract: oocytes were collected from unmated mice on day 1 (at 18 h post-hCG); zygotes were collected on 18 or 22 h post-hCG and staged according to the maturation of their pronuclei (PN1 being the least and PN5 the most mature stages of zygotic maturation prior to syngamy (Adenot *et al*. 1997)); two cell (42 h post-hCG); four cell (52 h post-hCG); eight cell (60 h post-hCG); morula (72 h post-hCG); blastocyst (90 h post-hCG), and late blastocyst (98 h post-hCG) stage embryos were collected directly from reproductive tract. After oocyte and zygote collection, any surrounding cumulus cells were removed by brief exposure to hyaluronidase (300 IU, Sigma).

### ICM isolation

The ICM was separated from the TE by immunosurgery (Solter & Knowles 1975). The blastocyst was freshly collected and had the zona pellucida removed by brief treatment with pronase (0.5% w/v) (Roche). They were then incubated with anti-mouse lymphocyte serum (1:10, Sigma, M4529) at 37 °C for 15 min, washed with HEPES-HTF three times and incubated with 1:10 diluted guinea-pig complement (Sigma, s1639) at 37 °C for 15 min. The lyzed TE cells were removed from the blastocyst by tituration with a narrow bore glass pipette. The isolated ICM was thoroughly rinsed and then immediately fixed for immunofluorescence staining. In some experiments the isolated ICM was disassociated into individual cells by gentle tituration in Ca^2^^+^/Mg^2^^+^ free PBS containing 0.25% (w/v) trypsin.

### Blastocyst outgrowths

Blastocysts were collected from the uterus and cultured in KSOM+AA media for 24 h, and then placed on a (acid washed) round cover glass fitted inside each well of a 4-well plate overlayed with 0.5 ml of DMEM/high glucose media (Thermo Scientific, SH3024301) supplemented with 100 μM β-mercaptoethanol (Sigma), 1× of MEM-NE amino acids solution (Invitrogen), 50 U/ml of penicillin (Sigma), 50 μg/ml of streptomycin (Sigma), and 10% of heat-inactivated ES cell qualified fetal bovine serum (Invitrogen) for 48 h. The cover glass was then removed and thoroughly washed in PBS and fixed immediately for immunofluorescence staining.

### Immunolocalization analysis

Immunofluorescence staining of embryos was performed as previously described (Li & O'Neill 2012, 2013). Embryos were collected directly from the reproductive tract, except where otherwise indicated, and after washing in PBS they were fixed at room temperature in 4% (w/v) formaldehyde (prepared fresh daily from paraformaldehyde) for 30 min and then permeabilized in 0.5% (v/v) Tween 20 and Triton X100 in PBS at room temperature for 40 min. They were then blocked in 30% (v/v) heterologous serum at 4 °C overnight followed by antigen retrieval methods. Unmasking of the 5meC and 5hmC antigens was performed by either brief exposure to 4 N HCl for 10 min (to denature DNA) or a combination of acid denaturation and tryptic digestion (0.25% (w/v) trypsin at 37 °C for 40 s (Invitrogen). Digestion was stopped by washing in 10% serum followed by extensive washing. Tryptic digestion was performed to remove proteins that may mask the epitopes within DNA. In one experiment the time and concentration of HCl or Trypsin was varied and the details are shown in the description of that experiment.

The conditions used for optimal detection of 5meC, 5hmC, and methyl-CpG-binding domain 1 (MBD1) staining were as previously described (Li & O'Neill 2012, 2013). Primary antibodies used were mouse anti-5meC MAB (AbD Serotec, Raleigh, NC, Clone 33D3); rabbit anti-5hmC antibody (Active Motif, Carlsbad, CA, USA, Cat#39769); rabbit polyclonal antibody to MBD1 (1:100 dilution; Abcam, Cambridge, UK, ab3753); rabbit anti-NANOG polyclonal antibody (1:100 dilution, Abcam, ab80892); rabbit anti-undifferentiated embryonic cell transcription factor 1 (UTF1) polyclonal (1:100 dilution, Abcam, ab24273); mouse anti-caudal-type homeobox 2 (CDX2) MAB (1:100 dilution, BioGenex, MU392A); goat anti-human GATA6 polyclonal antibody (1:70 dilution, R & D Systems, AF1700); rabbit anti-eomesodermin (EOMES) polyclonal antibody (1:100 dilution, Abcam, ab23345); monoclonal mouse anti-DNA methyltransferase (DNMT) 3A (IMG-268A, 1:1000, Imgenex, CA, USA), monoclonal mouse anti-DNMT3B (IMG-184A, 1:150, Imgenex, USA); rabbit polyclonal anti-DNMT1 (ab19905, 1:100, Abcam). Several types of secondary antibodies were used separately or in combination in this study depending on experimental design, including sheep anti-mouse IgG (FITC labelled 1:300 dilution, Sigma), goat anti-rabbit immunoglobulin (Ig)G (FITC labelled 1:250 dilution, Sigma; or Alexa Fluor 633, red, Molecular Probes, A21071; or Alexa fluor 405,blue, Molecular Probes, A31556), and donkey anti-goat IgG (1:250 dilution, Alexa Fluor 633, red, Molecular Probes, A21082). DNA was counter stained with propidium iodide (PI, 5 μg/ml) and mounted with VECTASHIELD mounting medium or directly mounted with ProLong Gold antifade reagent with DAPI (Molecular Probes, P36935).

Optical sectioning was performed with a Leica TCS SP5 confocal microscope equipped with a 63× oil objective (1.4 NA, optical section was 0.773 μm) for embryos from one cell to blastocyst or with a 40× oil objective (1.25 NA, optical section was 0.959 μm) for blastocyst outgrowths. For each experiment and replicate non-immune Ig control staining was performed as negative controls. Microscope and imaging settings were such that no signal was detected from these negative controls. Image analysis was performed with Image Pro-plus 6.3 (Media Cybernetics, Inc, Rockville, MD, USA). Three-dimensional (3D) compilations were created by Imaris 7.3 (BITPLANE) or ImageJ (National Institute of Health, Bethesda, MD, USA) and assembled with Adobe Illustrator CS6. In all experiments, the results are representative of three replicates with ten embryos or cells per treatment per replicate.

## Results

**Figure 1:**
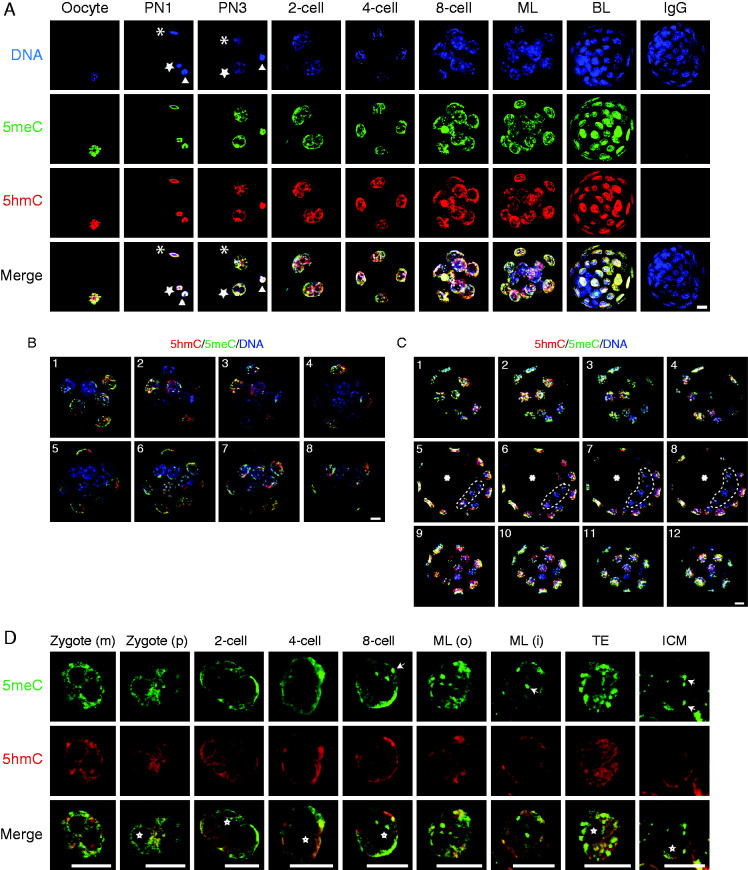
Patterns of 5meC and 5hmC staining in the oocyte and different stages of preimplantation embryo development. (A) z-stack projection images of staining for DNA (DAPI, blue), 5meC (green), 5hmC (red), and their merged (merge) images in the oocyte; zygote (PN1 and PN3); 2-cell, 4-cell, and 8-cell embryos; morula (ML); blastocyst (BL); and a BL stained with non-immune control IgG. (*) indicates the decondensing sperm head (in newly fertilized oocyte) or the male pronucleus (in PN3 zygote). (open star) indicates a female pronucleus. (open triangle) indicates the extruded polar body. (B) Single confocal optical sections through morulae triple stained for 5meC (green), 5hmC (red) and DNA (blue). The images show eight sequential sections taken at 0.88-μm intervals taken through morulae (1–8). (C) Single confocal optical sections through a blastocyst triple stained for 5meC (green), 5hmC (red), and DNA (blue). The images are sequential sections at 1-μm intervals at the lower pole (1–4, top row), through the equator (5–8, middle row), and upper pole (9–12, bottom row) of the embryo. Cells in the upper and lower poles are TE cells, the equatorial sections show the ICM (ICM enclosed by dotted line) encircled by the TE. (*) indicates blastocoel cavity. (D) Single confocal optical sections of staining of 5meC (green), 5hmC (red), and both merged in the individual maternal pronucleus (zygote (m)); paternal pronucleus (zygote (p)), and nuclei from two cell, four cell, eight cell embryos, and the outer and inner cells of morula (ML (o), ML (i)); and the TE and ICM of blastocysts. White arrows show representative examples of 5meC-intense staining foci; white *shows examples of nucleoli precursor bodies or nucleoli in late stage embryos. All scale bars, 10 μm.

Meiosis II chromosomes of the mouse oocyte (Fig. 1A) were heavily stained by both anti-5meC and anti-5hmC. Immediately following fertilization (the PN1 stage) the female pronucleus and the decondensing sperm head within the oocyte stained for both 5meC and 5hmC (as did the recently expelled second polar body). We confirmed earlier analyses that showed that 5meC and 5hmC persisted in both pronuclei as the zygotic cell-cycle progressed (Li & O'Neill 2012, Salvaing *et al*. 2012), and that the global levels of 5meC and 5hmC also persisted in each nucleus of 2-cell, 4-cell and 8-cell embryos (Fig. 1A).

At the late 8-cell stage the embryo undergoes a process of compaction whereby all the cells form tight associations with their neighbours. As cell proliferation progresses cells take up either an inner or outer position, creating the first opportunity for cells within the embryo to receive differential positional information. In the morula, the outer cells showed similar levels of 5meC and 5hmC as in earlier stages of development, but there was less staining in the nuclei of inner cells (Fig. 1A). The outer cells of the morula became committed to forming the multipotent TE lineage and the inner cells form the pluripotent ICM. In blastocysts these two morphologically distinct populations showed different immunodetectable levels of both 5meC and 5hmC; there was a marked reduction in the detectable levels of both antigens within the cells of the ICM while both antigens remained high within the TE (Fig. 1A).

The 3D structure of the morulae and blastocyst can make it difficult to visualize difference in staining levels between the inner and outer cells of the embryo. Thus, eight optical sections through the morulae were obtained, and these showed that cells on the periphery of the embryo had greater 5meC and 5hmC staining intensity than did those that appeared to be in an interior position (Fig. 1B). Twelve single confocal optical sections at different positions through the blastocyst are also shown (Fig. 1C). Four of these were sequential sections at the highest pole of the embryo, four were at the equatorial plane and four were at the lowest pole. The sections at the highest and lowest plane consisted entirely of TE cells. The equatorial sections showed a layer of TE cells that enclosed the fluid filled blastocoel cavity and a group of ICM cells. The optical sections showed that TE cells were heavily decorated with both 5meC and 5hmC and confirm that ICM cells had markedly less 5meC and 5hmC staining than the surrounding TE cells.

In the images comparing each stage of development (Fig. 1A) the power and image capture settings were held constant for all stages of development to allow for the assessment of the relative changes in 5meC and 5hmC levels. The large dynamic range of staining meant that the settings that allowed the low levels of staining in the cells of the ICM to be captured caused saturation of the signal at some other stages. To allow visualization of the patterns of staining at each stage of development, individual nuclei are shown in which image capture conditions were modified at some stages to avoid saturation (Fig. 1D). This means that these images should not be used for making between stage comparisons of the level of staining, although they do allow qualitative observations of changes in the intranucleoplasmic localization of each antigen. Single equatorial confocal sections are shown for representative nuclei at each stage of pre-implantation embryo development. 5meC staining was not uniform across the nucleoplasm; rather it showed areas of more concentrated staining. In the 1-cell through to the 4-cell stage, these areas tended to be at the periphery of the nucleus, and from the 8-cell stage onward 5meC staining was more localized to a number of intense chromocenters scattered throughout the nucleoplasm. In the inner cells of the morula and blastocyst, most 5meC staining was restricted to these chromocenters (examples are marked with white arrows, Fig. 1D). By contrast, 5hmC usually showed a more uniform pattern of staining across the nucleoplasm. Both antigens were excluded from the vesicular nucleoli precursor bodies and nucleoli present within each nuclei (examples are marked with *, Fig. 1D).

**Figure 2:**
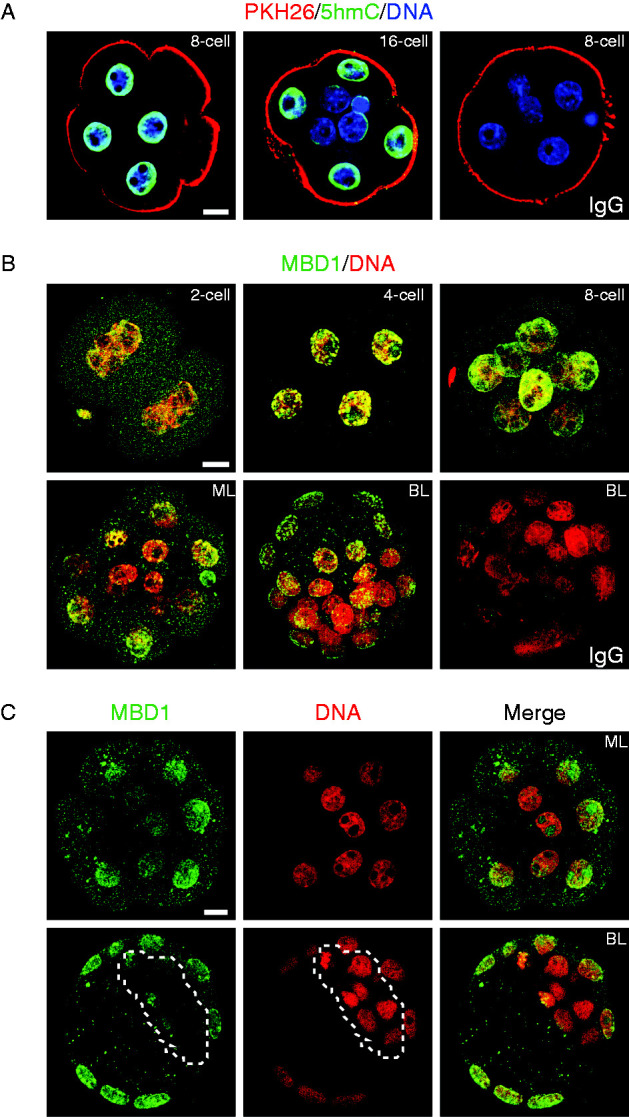
The relationship between the cell location and staining of 5meC, 5hmC, and MBD1 in preimplantation stage embryos. (A) Identification of the position of cells in 8-cell and 16-cell embryos by membrane labeling with PKH26. Live 8-cell and 16-cell embryos were first stained with the membrane label PKH26 (red). This stain labels the membranes exposed to the external fluid environment while the membrane of cells on the interior of post-compaction embryos are left unstained. This provides a means for unequivocal identification of cells on the inner and outer regions of the embryo. Embryos were then fixed and stained for 5hmC. (B) z-stack projection images showing merged MBD1 (green) and DNA (PI, red) in 2-cell, 4-cell, and 8-cell embryos; ML; BL; or non-immune IgG in BL. (C) Single equatorial sections of staining of MBD1 (green) and DNA (PI, red) and the merged staining of morula (ML, top row) and blastocyst (BL, bottom row) stage embryos. Scale bar=10 μm.

It can be difficult to clearly identify inner cells of the compacted embryo. Therefore an established lineage tracing strategy (Suwinska *et al*. 2008), which allows unequivocal discrimination of inner and outer cells was applied. This strategy uses an impermeant cell membrane labelling dye, PKH26 (Fig. 2A) which marks all of the cells in the uncompacted 8-cell embryo, because all of these cell are exposed to the media environment and hence the dye. After compaction and one round of cell division, some cells take up an inner position and are not directly exposed to the media environment and therefore can't take up the dye onto their membranes. Therefore, in the post-compaction embryo, the apical membranes of outer cells are labelled while the inner cells remain unlabelled by PKH26. These embryos were not subjected to tryptic digestion since we found that this caused degradation of PKH26 staining. 5hmC detection is independent of trypsin treatment (Li & O'Neill 2013) and since changes in 5hmC track the changes in 5meC (Fig. 1A) this was used as a proxy measure of methylation in these two cell populations. Equatorial optical sections of embryos were performed and these showed that in uncompacted 8-cell embryos all cells stained for PKH26 and had relatively homogeneous levels of 5hmC staining. In contrast, the inner cells (no PKH26 membrane staining) had lower 5hmC staining than outer cells (PKH26 positive on apical membranes) in post-compaction embryos (Fig. 2A).

To gain an alternative assessment of methylation levels, embryos were stained for MBD1 (Fig. 2B). This is a 5meC specific binding protein and is used as a proxy measure of 5meC (Yamazaki *et al*. 2007, Jin *et al*. 2010). Staining of each stage of development with this marker showed that there was some low level cytoplasmic staining but clear nuclear accumulation of MBD1 staining in 2-cell and 8-cell stage embryos. This staining was reduced in the inner cells of the morula and blastocyst (Fig. 2B). Equatorial confocal sections of the morula and blastocyst are also shown (Fig. 2C) and clearly indicate that the inner cells and ICM of morula and blastocysts respectively, had lower levels of nuclear MBD1 staining.

**Figure 3:**
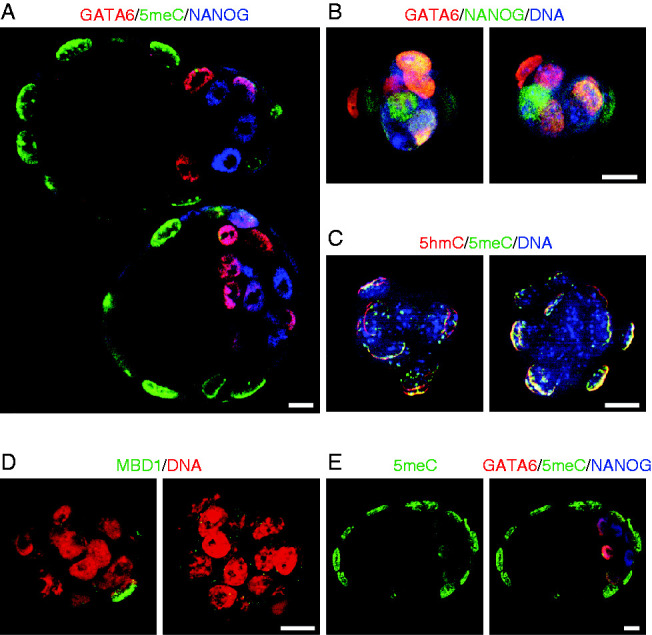
Further validation of differential global loss of methylation from the ICM. (A) Identification of the ICM by NANOG and GATA6 staining. Early blastocysts were triple stained for NANOG (blue), GATA6 (red), and 5meC (green). The merged image is a single equatorial confocal section of two blastocysts. (B) Two isolated ICM stained for NANOG and GATA6. ICMs were isolated by immunosurgery and triple stained for NANOG (green), GATA6 (red), and DNA (DAPI, blue). The images are z-stack projections. (C) Merged z-stack projection of two isolated ICM stained for 5meC (green), 5hmC (red), and DNA (DAPI, blue). (D) z-stack projection of two isolated ICMs stained for MBD1 (green) and DNA (red). (E) A single equatorial confocal section of a late stage blastocyst (98 h) showing either 5meC staining alone or a merged image (NANOG (blue), GATA6 (red), and 5meC (green)). Image shows the resolution of the NANOG and GATA6 staining cells into distinct populations. All bars=10 μm.

To further confirm the identity of hypomethylated cells within the blastocyst, we next stained blastocysts for canonical markers of the ICM lineage (NANOG and GATA6) and co-stained for 5meC (Fig. 3A). In early blastocysts (90 h post-hCG) the ICM showed the expected characteristic mosaic pattern of NANOG and GATA6 staining but these cells had little immunodetectable 5meC staining, whereas the outer TE cells were devoid of NANOG and GATA6 but were heavily decorated by anti-5meC (Fig. 3A). TE forms an epithelial-like layer, so in order to test whether this reduced the access of anti-5meC antibody to the cells of ICM, we next isolated ICM (by the immunosurgical removal of TE). The resulting naked ICM still showed the expected mosaic pattern of staining for NANOG and GATA6 (Fig. 3B), as well as the relatively low levels of 5meC and 5hmC staining (Fig. 3C). There was also a low level of MBD1 staining in these cells (Fig. 3D). In late blastocysts (98 h post-hCG), the ICM began to resolve into an outer layer of GATA6-positive presumptive hypoblast cells and an inner cluster of NANOG-positive epiblast cells. Both these populations were hypomethylated relative to the TE, while the GATA6-positive cells had higher 5meC levels than the NANOG-positive cluster (Fig. 3E).

**Figure 4:**
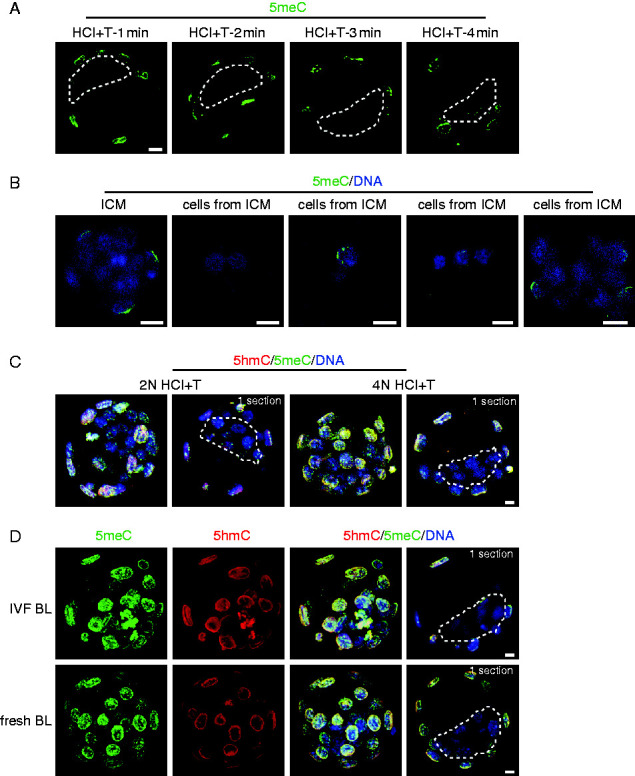
(A) Single equatorial confocal sections of 5meC (green) staining after epitope retrieval with brief acid exposure and then tryptic digestion for increasing times (1–4 min). The dotted line encloses the ICM in each section. (B) z-stack projection of 5meC (green) and DNA (DAPI, blue) in an intact ICM isolated by immunosurgery (ICM) and groups of disassociated cells from immunosurgically isolated ICM (cells from ICM). (C) Comparison of the use of lower concentration acid for a longer time with the conventional epitope retrieval approach. Images are z-stacks of the entire embryo and an individual equatorial confocal section. (D) Early blastocysts produced by IVF and then cultured (IVF BL) or blastocysts collected directly from the reproductive tract (fresh BL) were stained for 5meC and 5hmC. Images are z-stacks of the entire embryo and an individual equatorial confocal section (1 section). All bars=10 μm.

To assess whether the apparent loss of 5meC staining in the ICM was a result of further compaction of chromatin that may potentially cause epitope masking, we progressively increased the tryptic digestion period from 1 to 4 min. This had no effect on detection of 5meC in the TE while the ICM remained relatively poorly stained (Fig. 4A). Digestion beyond 4 min caused such extensive denaturation of chromatin that processing of the cells for staining became unmanageable. We noted that the cells of the ICM were very tightly compacted so to ensure that the low staining level was not due to restricted antibody access, we disassociated the ICM into individual cells by incubating the live ICM in Ca^2^^+^/Mg^2^^+^ free media with trypsin. The individual ICM cells showed no increase in the level of 5meC staining (Fig. 4B). In some studies longer exposure of cells to lower concentrations of acid (2 N HCl for 30 min) (e.g., Santos *et al*. 2002) has been used to affect 5meC epitope retrieval. We found that this approach was not as effective in retrieving antigens, but it still showed differentially high levels of 5meC and 5hmC in the TE compared with the ICM (Fig. 4C). Many previous 5meC immunolocalization studies in the preimplantation embryo have used embryos that were created by IVF or subjected to prolonged culture. We therefore compared staining in blastocysts created by IVF with blastocyst collected directly from the uterus. In both cases the embryos displayed higher levels of 5meC and 5hmC in the TE cells and a relatively low levels of both antigens in the ICM (Fig. 4D). We conclude that the reduction in staining of 5meC in the ICM reflects a loss of this antigen from the emerging pluripotent lineage.

**Figure 5:**
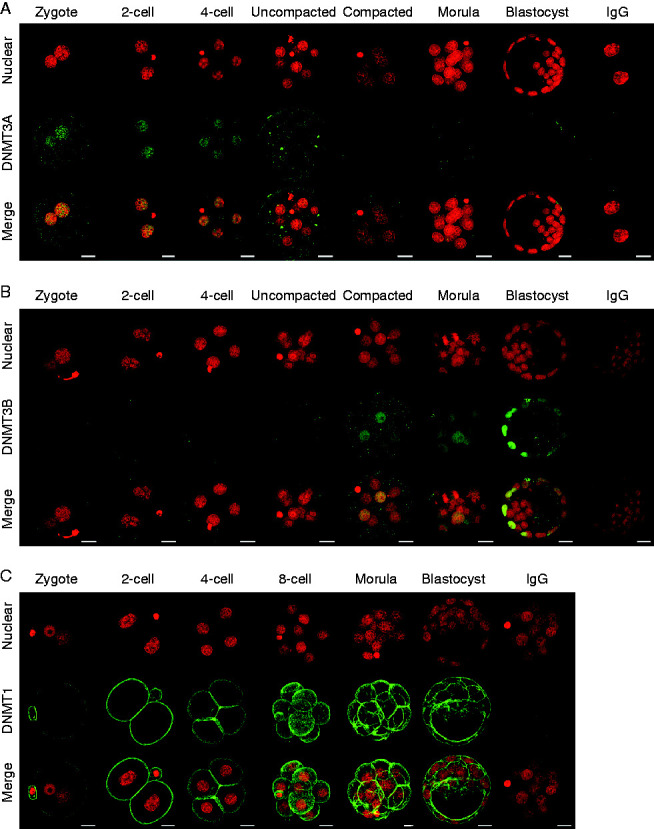
Staining of DNMT in the preimplantation embryo. Representative images of staining of DNA (PI, red) and (A) DNMT3A, (B) DNMT3b, and (C) DNMT1 (all green); and the merged image of these in zygotes, 2-cell, 4-cell, uncompacted 8-cell (uncompacted), compacted 8-cell (compacted); morula and blastocyst stage embryos. Images from the two channels were merged to show co-localization (merge). Images were z-stack compilation of sequential multiple equatorial sections. Non-immune IgG staining at the A) two cell; (B) blastocyst and, (C) morula stages are shown. Scale bars=20 μm.

**Figure 6:**
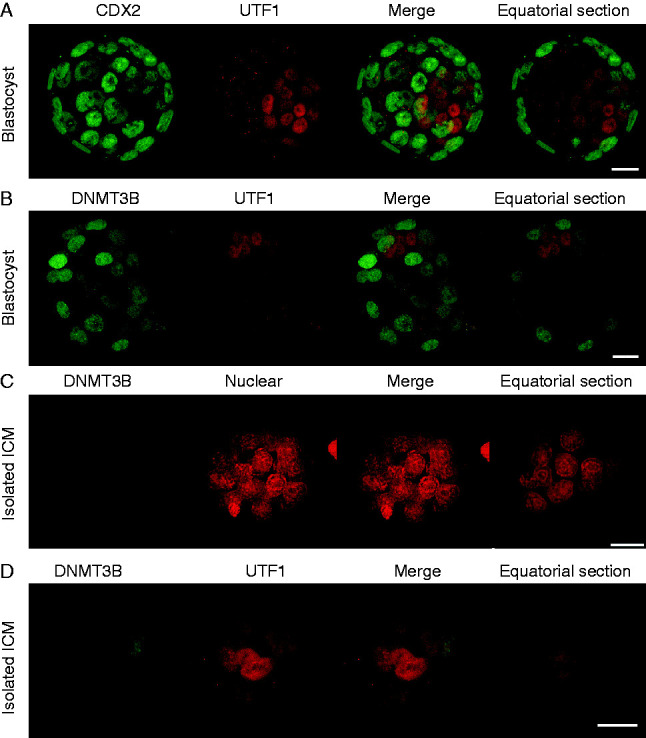
Confirmation of preferential localization of DNMT3B to TE in blastocysts. (A) Early blastocysts were stained for markers of the TE (CDX2, green) and ICM (UTF1, red). (B) Early blastocysts were stained for DNMT3B (green) and ICM (UTF1, red), z-stack projections and a single equatorial confocal section are shown. (C) Immunosurgically isolated ICM were stained for DNMT3B and counterstained with PI or (D) UTF1. All panels consisted z-stack projections and a single equatorial confocal section. Scale bars=20 μm.

The DNA methyltransferase family of enzymes is responsible for genome methylation. Nuclear DNMT3A was evident in both pronuclei of the zygote and in each nucleus of the 2-cell and 4-cell embryos (Fig. 5A). Its level was reduced in 8-cell embryos and was below the detection limit of the immunostaining technique in all cells of both the morula and blastocyst-stage embryo (Fig. 5A). In contrast, DNMT3B was below the assay detection limit in the nuclei of zygotes, 2-cell and 4-cell embryos (Fig. 5B). It was detected at a low level in the nuclei of uncompacted 8-cell embryos and at a slightly higher levels in the compacted 8-cell and morulae. There was little detectable DNMT3B staining in the ICM in blastocysts, while the TE showed high, but variable levels, of staining (Fig. 5B). DNMT1 was present in all preimplantation stages of development but was generally localized to the periphery of cells rather than their nucleus (Fig. 5C). Of the major classes of DNMTs, DNMT3B showed a pattern of differential localization most consistent with the low levels of 5meC in the ICM relative to the TE. UTF1 staining clearly discriminated the ICM from the CDX2-positive TE (Fig. 6A), yet little nuclear DNMT3B could be detected in the UTF1-postive ICM (Fig. 6B). To exclude the possibility that the absence of ICM staining was due to the TE preventing adequate antibody penetration, the ICM was isolated by immunosurgery and then stained for DNMT3B. The isolated ICM still showed little staining for DNMT3B (Fig. 6C) but was readily stained for UTF1 (Fig. 6D).

**Figure 7:**
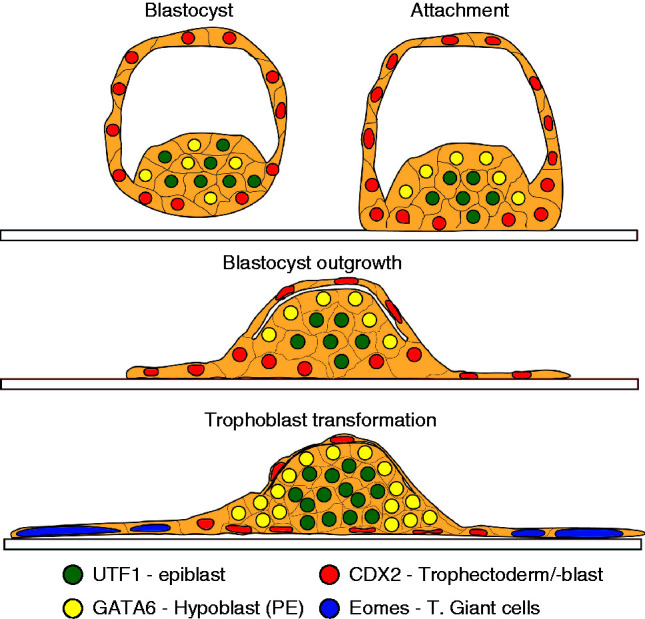
A diagrammatic representation of the changes in blastocyst morphology during blastocyst outgrowth over 48 h cultures of blastocysts *in vitro*. The formation and allocation of cells to each of the cell populations defined by the lineage markers are shown. These structures correspond to the confocal images shown in Fig. 4.

**Figure 8:**
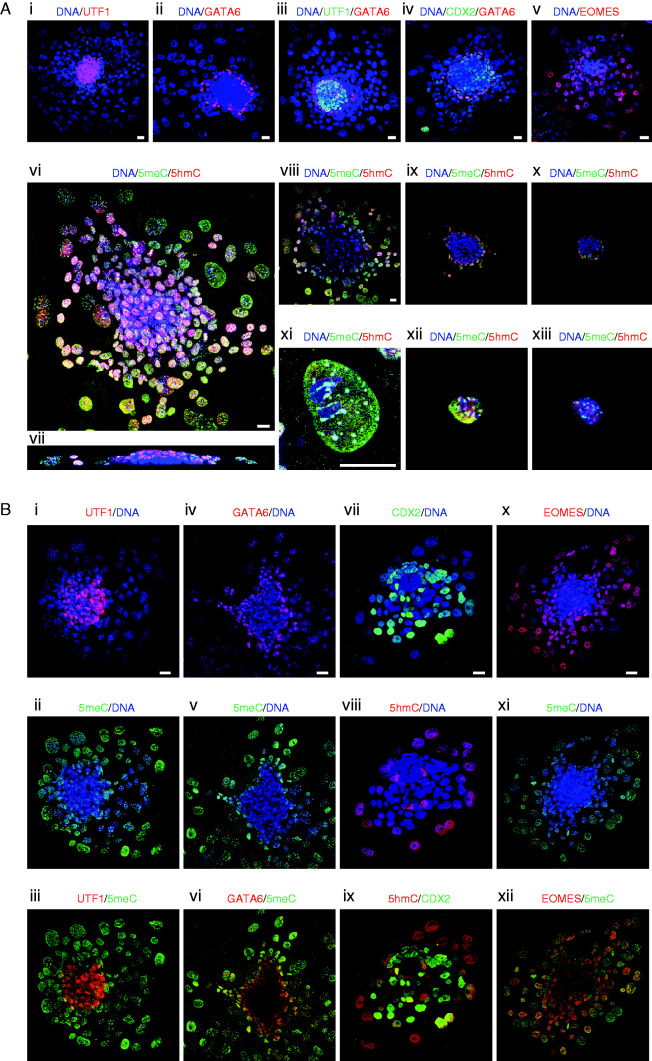
Changes in 5meC and 5hmC staining in each lineage formed during implantation *in vitro*. (A) Panels i, ii, iii, iv and v are z-stack projections of 48 h blastocyst outgrowths and show the localization and distribution of each lineage formed during outgrowth. All cells were stained for DNA (DAPI, blue) and for the lineage markers: i) epiblast (UTF1, red); ii) hypoblast (GATA6, red); iii) epiblast (UTF1, green), and hypoblast (GATA6, red); iv) trophoblast (CDX2-green) and hypoblast (GATA6, red); and v) TGCs (EOMES, red). vi, vii, viii, ix, x, xi, xii, xiii, xiv) staining of outgrowths for 5meC (green), 5hmC (red), and DNA (DAPI, blue) of a vi) z-stack projection in the x–y dimension and vii) cross-section in x–z dimension; viii, ix, x) single confocal sections at progressive heights along the z-axis through the embryo shown in vi. xi, xi, xii, xiii) Higher power images of individual xi) TGC, xii) small TB, and xiii) hypoblast cells respectively. (B) Blastocyst outgrowths co-stained for 5meC or 5hmC and markers for each lineage. Each panel shows a z-stack projection in the x–y dimension of representative 48 h blastocyst outgrowths. Merged images of staining for lineage markers and cytosine modifications: epiblast i) UTF1 (red) with DNA (blue), ii) the same embryo stained for 5meC (green) and DNA, iii)UTF1 (red) with 5meC (green); hypoblast iv) GATA6 (red) with DNA (blue), v) 5meC (green) with DNA, vi) GATA6 (red) with 5meC (green); trophoblast vii) CDX2 (green) with DNA (blue), viii) 5hmC (red) with DNA (blue); TB ix) CDX2 (green) with 5hmC (red); TGCs x) EOMES (red) with DNA, xi) 5meC (green) with DNA (blue), xii) EOMES (red) with 5meC (green). All scale bars=20 μm.

Further development of the blastocyst normally requires implantation into the uterine endometrium. A process that shows some analogy to implantation can be achieved *in vitro* by the continued culture of blastocysts in complex media on a suitable substratum (blastocyst outgrowth, illustrated in Fig. 7). The TE cells of the early blastocyst do not have adhesive properties, but by the late blastocyst stage these cells become adhesive to their substratum and upon adhesion begin to develop invasive growth patterns. We used this change in property to distinguish between TE cells and the formation of trophoblast cells (TBs). *In vitro* the late blastocyst hatches from its surrounding zona pellucida and attaches to its substratum. Upon attachment, the blastocoel cavity progressively collapses, the TB proliferates and some of these cells grow out onto the substrate surface and undergo endoreduplication to form trophoblast giant cells (TGCs). The ICM continues its segregation into distinct epiblast and hypoblast (also known as the primitive endoderm) populations. The resulting embryos are composed of an inner cluster of pluripotent epiblast cells (UTF1-positive) at their core (Fig. 8A i, iii), surrounded by a layer of hypoblasts (GATA6-positive) (Fig. 8A ii and iii). This inner core of epiblast and hypoblast is overlayed by a sheet of TBs (CDX2-positive) which extends beyond the margins of the epiblast/hypoblast mass (Fig. 8A iv) and differentiates into the TGCs (EOMES-positive). These cells are readily identified by their giant nuclei (Fig. 8A v).

Considerable heterogeneity in the levels of nuclear 5meC and 5hmC was detected across the cell population of blastocyst outgrowths (Fig. 8A vi, vii, viii, ix and x). The most noticeable feature was that the presumed epiblast (inner population of cells) had little 5meC or 5hmC staining (Fig. 8A vii, viii, ix and x) compared with the overlaying TB cells, which stained heavily for both epigenetic modifications (Fig. 8A vi). Figure 8A xi, xii and xiii shows higher resolution images of a representative TGC, small TB, and a hypoblast cell respectively. Distinctive distribution patterns of 5meC and 5hmC across the nucleoplasm are noted in each cell type. To definitively relate these changes to the various cell types within the embryo, outgrowths were co-stained with markers of each lineage and 5meC or 5hmC. The UTF1-positive epiblast cells coincided with the population with low staining of 5meC (Fig. 8B i, ii and iii). Many GATA-6-positive hypoblast cells had 5meC staining, but heterogeneity in staining levels was observed (Fig. 8B iv, v and vi). Those that were more internal to the embryo and closest to the epiblast were relatively hypomethylated while those furthest from the epiblast showed progressively higher levels of global methylation. We found that the CDX2-antigen was unstable after brief tryptic digestion, so for the CDX2-positive TB we assessed the level of 5hmC (which does not require tryptic digestion for epitope retrieval (Li & O'Neill 2013)) (Fig. 8B vii, viii and ix). This showed that the TB cells overlaying the epiblast and hypoblast stained heavily for 5hmC (and by extrapolating from Fig. 8A vi, we can say that this population would also be positive for 5meC). The TGCs were generally CDX2-negative, but were also marked by anti-5hmC. Both the TGCs and most TBs were EOMES-positive and stained heavily for 5meC (Fig. 8B x, xi and xii). This analysis shows that the epiblasts maintained a low level of methylation and hydroxymethylation, and that the GATA6-positive hypoblast population had higher, but heterogeneous levels of 5meC and 5hmC after blastocyst outgrowth. Each of the first differentiated lineages in the early embryo showed elevated levels of both 5meC and 5hmC, while the modifications were maintained at a low level in the pluripotent lineage.

## Discussion

This study provides the first simultaneous mapping of the global levels of 5meC and 5hmC epigenetic modifications in each cell of the embryo from fertilization through the first several rounds of differentiation. It shows that highly dynamic global changes in the global levels of solvent exposed 5meC and 5hmC occur during important developmental transitions in the early mouse embryo. The most profound change was a progressive loss of staining of both epigenetic modifications in the cells committed to the pluripotent ICM lineage, resulting in markedly different global levels of methylation and hydroxymethylation in the first two cell lineages generated in the early embryo, the TE and ICM. Our results show that the hypomethylated state of ICM persisted across the first round of differentiation into the epiblast, while differentiation into the hypoblast was accompanied by an increase in both 5meC and 5hmC that was heterogeneous between cells. In contrast, the hypermethylated state of the TE persisted as these cells differentiated into TBs and TGCs *in vitro*.

Rearrangement of the 5meC staining pattern within the nucleoplasm occurred during the preimplantation stage. These changes in localization of staining, together with dynamic changes in the size and conformation of the nuclei challenge conventional forms of quantification of staining levels. Therefore, valid quantitative analysis across stages of development was not possible, so relative changes in staining levels and patterns were compared. To our knowledge, a genome-wide base-level analysis of mouse zygotes has not previously been performed, but reduced representation bisulfite sequencing of <5% of the genome showed that the levels of methylation were similar in the maternal and paternal genomes of the zygote, and that this level was similar to that in the oocyte, but less than in sperm (Smith *et al*. 2012, Guo *et al*. 2013). This method is biased towards the quantitatively minor CpG enriched regions of the genome yet it showed a reduction in the methylation of the paternally inherited genome to a level similar to that of the maternal DNA by the PN2 stage (Guo *et al*. 2013) and the methylation levels in the zygote and 2-cell embryo were similar (Smith *et al*. 2012). Whole genome-wide methylation analysis of the 2-cell embryo confirmed a similar (although slightly lower) average methylation level in the 2-cell embryo compared with the oocyte. A further small decrease occurred in 4-cell embryos and a large decrease in the methylation levels of cells of the ICM (Wang *et al*. 2014). A decrease in methylation of the blastocyst was confirmed by deep-hairpin bisulfite sequencing of a small number of CpG dyads, although this analysis did not discriminated between the ICM and TE (Arand *et al*. 2015). Since immunolocalization is not a strictly quantitative tool, it is unlikely to be capable of reliably detecting the relatively small changes in average methylation levels detected by genome-wide base level analysis of the 2-cell and 4-cell embryo. The marked change in average methylation of the ICM found by base-level analysis (Wang *et al*. 2014) is consistent with the low levels detected by our immunolocalization analysis. A wide range of experiments were undertaken to confirm that this reduced staining reflected changes in the levels of the antigen in the ICM. We have also recently shown that the immunolocalization methods used in the present study can readily detect the expected approximately 50% reduction in global methylation levels resulting from treating 2-cell embryos with DNMT inhibitors for one cell cycle (Li & O'Neill 2012). An earlier study that showed high levels of 5meC immunostaining in the ICM compared with the TE (Santos *et al*. 2002) used IVF embryos and a different process of acid denaturation (2 N HCl for 30 min) from that used in the present study, yet we found a similar pattern of hypermethylation of TE and hypomethylation of the ICM, whether embryos were obtained by IVF or collected from the reproductive tract, and this was also the case when a lower acid concentration and longer incubation times were applied for epitope retrieval.

The difficulty in isolation of sufficient quantities of purified TE cells has to date prevented whole genome base-level analysis of the TE but this is clearly a priority as sensitivity of analysis methods improves. We are not aware of any whole-genome base level comparison of the methylation in the TE and ICM. An analysis of methylation-sensitive restriction analysis of mouse trophoblast stem cells and ES cells identified a number of differentially methylated regions that were generally hypermethylated in the mouse trophoblast stem cells compared with ES cells. In contrast, an analysis of a subset of these sites within blastocysts showed relative hypomethylation in both the ICM and the TE compared with the stem-cell lines (Nakanishi *et al*. 2012), indicating possible fundamental epigenetic differences between stem-cell lines and their embryonic counterparts. Whole-genome base-level analysis showed that the change in methylation of the ICM was most evident in 3′-UTRs, introns, and exons. There was little change in methylation levels of 5′-UTRs and CpG islands, and promoters were generally hypomethylated across development, showing only modest levels of remodelling as a class (Wang *et al*. 2014). The marked differences in methylation reprogramming observed between different functional genetic elements (Wang *et al*. 2014) point to the dangers of extrapolating from observations made at selected loci or elements to broader epigenetic mechanisms.

Whole-genome analysis showed that while the relative methylation levels of CpGs in the ICM was 0.2, this increased to 0.73 in the E7.5 whole embryo (Wang *et al*. 2014), and a similar finding was made using reduced representation bisulfite sequencing (Smith *et al*. 2012). These analyses were performed on the whole embryo and thus do not account for potential differences between the various cell lineages within these embryos. The current study shows a lineage specific analysis of 5meC and 5hmC in the blastocyst outgrowth model of post-implantation development for the first time. The hypomethylated state of the epiblast after outgrowth is consistent with the low level of methylation levels of ES cell lines held in the pluripotent ground state (Leitch *et al*. 2013). The GATA6-positive hypoblast lineage showed increased global 5meC staining in late blastocysts, and this persisted in blastocyst outgrowths. Staining of post-implantation paraffin sectioned embryos (E7.5) showed considerable heterogeneity in staining levels. By this stage the embryo has undergone gastrulation, hence much of the epiblast has differentiated into the primary germ layers. Chemical analyses showed that the 5meC levels of the combined extraembryonic lineages were lower than those of the combined embryonic lineages (Senner *et al*. 2012). The methodology described in the present study provides a suitable platform for examination of each emerging cell lineage in the post-implantation embryo *in utero*.

Early studies in the field showed that the net DNA methylase activity is high and stable across the first three cleavage divisions to the 8-cell stage but then declined by the blastocyst stage (Monk *et al*. 1991). The localization of DNMT1 in the embryo is controversial. DNMT1 alone is reported to be sufficient to account for maintenance of imprinted loci (Hirasawa *et al*. 2008). Some reports show that the somatic form of the enzyme is located in the nucleus at each stage of preimplantation development (Kurihara *et al*. 2008) in all stages, with the exception of the 1-cell stage (Cirio *et al*. 2008*a*, *b*), while other studies have identified it as having a primarily cytoplasmic localization at all stages (Ratnam *et al*. 2002, Hirasawa *et al*. 2008). Our current observations are most consistent with a predominately cytoplasmic localization. The basis for these conflicting observations remain to be explained and require further investigation. A primarily cytoplasmic location has been taken to infer an absence of activity and hence passive demethylation during early development (Grohmann *et al*. 2005, Morgan *et al*. 2005). However, the present study and others (Li & O'Neill 2012, Wang *et al*. 2014) did not find evidence of a pervasive loss of 5meC in the cleavage stage embryo (up to the 8-cell stage), which is not consistent with a generalized failure of maintenance methylation. Our observations of nuclear localization of DNMT3A in the early embryo followed by its loss and replacement with DNMT3B in the nuclei of all cells except the ICM, are consistent those of earlier reports (Hirasawa *et al*. 2008, Hirasawa & Sasaki 2009). Gene expression analysis showed that of a wide range of epigenetic modifiers tested *Dnmt3B (*and* Dnmt3L*) showed high levels of expression in the TE relative to the ICM (Burton *et al*. 2013). It has been argued that the upregulation of *Prdm14* expression in cells determined to the pluripotent lineage acts to repress expression of *Dnmt3B* and *Dnmt3L* in the ICM relative to the TE (Burton *et al*. 2013, Yamaji *et al*. 2013). A hypothesis for the observed changes in the global pattern of 5meC may be that from fertilization to the 8-cell stage DNMT3A is a important methylase activity in embryonic cells, and then from the 8-cell stage, DNMT3B may become a primary methylase and is progressively restricted to the outer cells of the embryo and the eventual TE cells. As the emerging hypoblast lineage began to resolve in the late blastocyst an increase in global 5meC was evident, which is consistent with existing evidence showing that DNMT3B also increased as this lineage differentiation begins (Hirasawa & Sasaki 2009). Hirasawa and Sasaki (2009) also found that increased DNMT3B was evident in later stage epiblasts (Day 5.5). The current study did not look at this stage of development although the epiblast in the outgrowth experiments is thought to be at a similar stage, but increased 5meC in the epiblast was not evident in these cells. It is clear that further detailed analyses are required to define the roles of DNMTs in regulating changes in methylation levels across key embryonic transitions.

Analysis of interphase nuclei showed that a large amount of the immunodetectable 5hmC staining did not coincide with 5meC staining; 5hmC usually showed more generalized staining across the nucleoplasm, while 5meC staining tended to have a more restricted focal pattern of localization. This was most evident in the emerging pluripotent lineage where 5meC staining was predominantly restricted to a number of intense staining foci. This may reflect the known strong association between 5meC and the repressed heterochromatic fraction of the genome (Celik *et al*. 2014), while it is beginning to be recognized that 5hmC is preferentially associated with the euchromatic fraction (Kubiura *et al*. 2012). Some studies (Gu *et al*. 2011, Iqbal *et al*. 2011, Zhang *et al*. 2012) have shown a marked preferential presence of 5hmC within the paternally-derived pronucleus of the zygote, yet this was not evident in the current analysis, or in other recent reports (Salvaing *et al*. 2012, Li & O'Neill 2013), which showed ample immunostaining of 5hmC in both pronuclei. It has been shown that low 5hmC staining of maternal pronuclei occurred when staining was performed in non-equilibrium conditions and that was sufficient to account for the differences in these reports (Li & O'Neill 2013). This finding of similar levels of 5hmC in both pronuclei is consistent with genome-wide base-level analyses which showed that 101,352 CpGs in the paternally inherited genome and 124,425 CpGs in the maternal line were enriched for 5hmC, although the average hydroxymethylation levels at these sites was higher in male than female DNA (Wang *et al*. 2014). Replication-dependent loss of 5hmC during preimplantation development has been proposed (Inoue & Zhang 2011) and this may be consistent with the asymmetric distribution of 5hmC between the Watson–Crick strands detected by whole genome analysis (Wang *et al*. 2014). TET-mediated demethylation of 5meC may have a role in remodelling some loci, such as *Oct4* and *Nanog* or specific genetic elements (Gu *et al*. 2011), but current evidence does not support a predominantly hyperhydroxymethylated state for male DNA and an hypohydroxymethylated state for female DNA in zygotes.

The present study and recent whole-genome base-level analyses indicate a number of differences in the pattern of reprogramming global cytosine methylation than has long been held to be the case. The results point to the commitment of cells to the pluripotent ICM being associated with global hypomethylation of the genome while the first rounds of lineage differentiation were associated with global hypermethylation. The present study provides a new basis for investigating the mechanisms that control epigenetic reprogramming in the early embryo.
